# Neutrophils and cancer immunotherapy: Potential mechanisms and challenges in breast cancer – a narrative review

**DOI:** 10.1097/MD.0000000000044831

**Published:** 2025-09-26

**Authors:** Emmanuel Ifeanyi Obeagu

**Affiliations:** a Department of Biomedical and Laboratory Science, Africa University, Mutare, Zimbabwe.

**Keywords:** breast cancer, cancer immunotherapy, neutrophil extracellular traps, neutrophils, tumor microenvironment

## Abstract

Neutrophils, the most abundant white blood cells in circulation, are gaining recognition for their intricate roles in cancer biology, particularly in breast cancer. These cells can exhibit dual behavior, either promoting tumor progression through immunosuppressive mechanisms or combating cancer by enhancing anti-tumor immune responses. Tumor-associated neutrophils (TANs) are highly plastic, with their phenotype and function influenced by the tumor microenvironment. This duality underscores the complexity of targeting neutrophils for therapeutic purposes in breast cancer immunotherapy. Key mechanisms such as neutrophil extracellular traps (NETs) and immune modulation by TANs are central to their role in breast cancer. NETs contribute to cancer metastasis by facilitating tumor cell adhesion and immune evasion, while TANs can suppress cytotoxic T-cell activity through the release of immunosuppressive mediators like arginase-1 and reactive oxygen species (ROS). Empirical studies have demonstrated that high neutrophil-to-lymphocyte ratios and excessive NET formation are correlated with poor prognosis in breast cancer patients, particularly those with aggressive subtypes such as triple-negative breast cancer.

## 1. Introduction

Breast cancer remains the most commonly diagnosed cancer in women worldwide, posing a significant health challenge due to its heterogeneous nature and high metastatic potential.^[1]^ Over the past few decades, advances in immunotherapy have revolutionized cancer treatment by harnessing the immune system to combat tumor growth.^[2]^ While much attention has been given to lymphocytes, particularly T cells, emerging evidence suggests that neutrophils also play a critical role in modulating the tumor microenvironment (TME).^[3]^ This dual role of neutrophils – as both tumor promoters and suppressors – makes them intriguing targets in breast cancer immunotherapy. Neutrophils, constituting 50 to 70% of circulating leukocytes, are the first line of defense against infections.^[4]^ In the context of cancer, they are recruited to the TME by tumor-derived factors such as granulocyte colony stimulating factor (G-CSF) and interleukin-8 (IL-8). Tumor-associated neutrophils (TANs) are classified into 2 phenotypes: anti-tumor (N1) and pro-tumor (N2), depending on signals in the TME. Empirical studies have revealed that high TAN infiltration is associated with poor prognosis in breast cancer, particularly in triple-negative breast cancer (TNBC) and HER2-positive subtypes mechanisms through which neutrophils influence breast cancer is via neutrophil extracellular traps (NETs). These web-like structures, composed of DNA and proteolytic enzymes, are released by activated neutrophils. NETs can promote metastasis by enhancing cancer cell adhesion and invasion. A study by Zhang et al^[5]^ demonstrated that NETs facilitate breast cancer cell seeding in distant organs by interacting with platelets and endothelial cells. Furthermore, ex models showed that DNase treatment to degrade NETs significantly reduced metastatic burden in TNBC mouse models.^[6–8]^

In addition to NETs, their pro-tumor effects through immunosuppressive mechanisms. They release reactive oxygen species (ROS), arginase-1, and matrix metalloproteinases (MMPs), which suppress T-cell activation and promote angiogenesis. A study involving 500 breast cancer patients revealed that elevated neutrophil-to-lymphocyte ratios (NLRs), a marker of systemic inflammation, were correlated with lower overall survival rates, particularly in advanced-stage patients. These findings underscore the imp neutrophil-mediated immune evasion in breast cancer progression.^[9]^ Conversely, neutrophils also possess anti-tumor capabilities under certain conditions. N1 TANs, which are polarized in the presence of interferons and other inflammatory cytokines, can secrete tumor necrosis factor-alpha (TNF-α) and interleukin-1 beta (IL-1β) to recruit cytotoxic T cells and enhance anti-tumor immunity. Preclinical studies have shown that boosting N1 TAN polarization through TGF-β inhibitors can enhance the efficacy of immune checkpoint blockade (ICB) in murine breast cancer models. These findings highlight the therapeutic pf reprogramming TANs to favor an anti-tumor phenotype.^[10]^ The role of neutrophils in breast cancer also varies across different subtypes. For instance, in HER2-positive breast cancer, neutrophils are implicated in trastuzumab resistance. A study by Bui et al found that TAN-derived ROS reduced antibody-dependent cellular cytotoxicity (ADCC) by impairing natural killer (NK) cell function. In contrast, TNBC tumors, which lack targeted thera, exhibit high neutrophil infiltration that contributes to aggressive disease progression. These subtype-specific differences highlight the need for therapeutic approaches.^[11]^ Despite the growing understanding of neutrophil involvement in breast cancer, several challenges remain. Neutrophils’ functional plasticity, influenced by both the TME and systemic factors, complicates efforts to selectively target their pro-tumor functions. Additionally, therapies aimed at reducing neutrophil activity must balance the risk of impairing their essential role in antimicrobial defense. Clinical trials targeting neutrophil-related pathways, such as CXCR2 antagonists and NET inhibitors, have shown promise but require further validation.^[12,13]^

This review aims to explore the multifaceted role of neutrophils using on their potential mechanisms of action and the challenges associated with targeting them in immunotherapy.

### 1.1. Aim

This review aims to explore the multifaceted role of neutrophils in breast cancer and their emerging potential as targets in cancer immunotherapy.

### 1.2. Rationale

Breast cancer remains one of the most prevalent and challenging cancers globally, with various subtypes that exhibit distinct biological behaviors, therapeutic responses, and prognoses.^[14]^ Traditional treatments, such as surgery, chemotherapy, and radiotherapy, have significantly improved survival rates, yet metastatic disease and therapy resistance continue to pose major hurdles. The advent of immunotherapy has revolutionized cancer treatment, but its full potential remains untapped, particularly in the context of breast cancer. As our understanding of the immune landscape of tumors deepens, neutrophils – once primarily viewed as passive participants in immune defense – have emerged as key players in cancer progression, immunity, and therapeutic response. Neutrophils are abundant in the tumor microenvironment (TME) and exhibit remarkable plasticity, with the ability to adopt either pro-tumorigenic or anti-tumorigenic roles depending on the cues they receive from the surrounding environment.^[15]^ This dual function has led to growing interest in neutrophils as potential therapeutic targets for cancer immunotherapy. Neutrophil-targeted therapies, such as reprogramming TANs to an anti-tumor phenotype, inhibiting their recruitment to tumors, or disrupting the formation of NETs, offer new avenues for improving cancer treatment, particularly in aggressive subtypes like TNBC, which are resistant to traditional therapies.

Their functional heterogeneity, the risk of disrupting their essential roles in immune defense, and the need for precise targeting to avoid adverse effects make neutrophil-based therapies complex and difficult to implement. Additionally, the tumor microenvironment itself – rich in immunosuppressive signals – complicates efforts to shift neutrophils from their pro-tumor phenotype to a tumor-fighting one. The rationale behind this review is to highlight the emerging role of neutrophils in breast cancer, provide an overview of the latest research on neutrophil-targeted therapies, and critically assess the challenges involved in translating these strategies into clinical practice. By consolidating current knowledge, this review aims to foster a deeper understanding of neutrophil biology and its potential as a therapeutic target, ultimately contributing to the development of more effective, personalized treatment strategies for breast cancer patients.^[15]^

## 2. Review methodology

This narrative review aimed to comprehensively examine the role of neutrophils in breast cancer, particularly in the context of immunotherapy. The methodology employed involved a systematic approach to gathering, analyzing, and synthesizing current literature to provide a comprehensive understanding of neutrophil involvement in breast cancer and its potential as a therapeutic target. Below is a detailed account of the methodology used in this review.

### 2.1. Literature search and selection criteria

The primary sources of information were peer-reviewed journal articles, clinical trial reports, and reviews available in major scientific databases, including PubMed, Google Scholar, Scopus, and Web of Science. Keywords such as “neutrophils,” “breast cancer,” “tumor-associated neutrophils,” “immunotherapy,” “neutrophil reprogramming,” “neutrophil extracellular traps,” and “immune modulation” were used in various combinations to ensure comprehensive coverage of the topic.

The selection of studies was based on the following inclusion criteria:

- Studies that focused on neutrophil function and its role in cancer progression, particularly in breast cancer.
- Research on the potential of neutrophil-based immunotherapy in treating breast cancer.
- Studies discussing the mechanisms by which neutrophils contribute to tumor immune evasion, metastasis, and therapy resistance.
- Preclinical and clinical studies that investigated the use of neutrophil-targeted therapies.

Exclusion criteria included:

- Studies that did not directly address neutrophils in the context of breast cancer or cancer immunotherapy.
- Non-peer-reviewed articles, abstracts, and conference proceedings without sufficient methodological details.

### 2.2. Ethical approval

Not applicable as this is a narrative review.

### 2.3. Potential mechanisms of neutrophil involvement in breast cancer

Neutrophils, long regarded as first responders in innate immunity, exhibit a dynamic role in breast cancer, intricately modulating the tumor microenvironment (TME). Their involvement spans both tumor-promoting and tumor-suppressing activities, making them key players in breast cancer progression and a potential target for therapeutic intervention.

### 2.4. Tumor-associated neutrophils (TANs)

Neutrophils recruited to the TME differentiate into TANs, which exhibit phenotypic plasticity influenced by local cytokines and growth factors. TANs are broadly categorized into pro-tumorigenic (N2) and anti-tumorigenic (N1) phenotypes. The tumor microenvironment, rich in factors like TGF-β, skews neutrophils toward the N2 phenotype, characterized by immunosuppression, promotion of angiogenesis, and facilitation of metastasis. Conversely, N1 TANs, promoted by type I interferons, produce inflammatory cytokines such as TNF-α and IL-1β, which recruit cytotoxic T cells and enhance anti-tumor immunity. Empirical evidence from preclinical studies highlights the potential of reprogramming TANs. In murine breast cancer models, TGF-β inhibitors successfully shifted TANs to the N1 phenotype, enhancing the efficacy of checkpoint inhibitors.^[16,17]^

### 2.5. Neutrophil extracellular traps (NETs)

NETs are web-like networks released by neutrophils to trap and neutralize invading pathogens. These structures are made of DNA strands coated with antimicrobial proteins such as histones, neutrophil elastase, and myeloperoxidase. The process by which neutrophils release NETs is called NETosis, a unique form of cell death distinct from apoptosis or necrosis. Although NETs serve as a defense mechanism in infections, increasing evidence links them to cancer progression. In breast cancer, NETs can promote tumor growth and metastasis by capturing circulating tumor cells and helping them anchor and invade distant tissues. In TNBC, preclinical studies show that degrading NETs using DNase enzymes can reduce metastatic spread, highlighting NETs as potential therapeutic targets. Moreover, NETs interact with platelets and blood vessel walls to form blood clots, creating a pro-thrombotic environment that further supports tumor dissemination.^[18,19]^

### 2.6. Immune suppression in the tumor microenvironment

Neutrophils contribute significantly to immune evasion in breast cancer by modulating the activity of other immune cells. They release immunosuppressive mediators such as arginase-1, ROS, and nitric oxide, which inhibit T-cell proliferation and impair NK cell activity. Clinical studies have shown that a high neutrophil-to-lymphocyte ratio (NLR), a surrogate marker of systemic inflammation, correlates with poor prognosis in breast cancer patients. In HER2-positive breast cancer, TANs have been linked to resistance to trastuzumab therapy by impairing NK cell-mediated ADCC through ROS production.^[20,21]^

### 2.7. Angiogenesis and tumor growth

Neutrophils promote tumor angiogenesis by releasing vascular endothelial growth factor and matrix metalloproteinases (MMPs). These molecules degrade the extracellular matrix, facilitating new blood vessel formation and tumor growth. In TNBC, high neutrophil infiltration has been associated with increased angiogenesis and enhanced tumor invasiveness. Therapeutic strategies targeting vascular endothelial growth factor or MMPs in conjunction with TAN modulation are being explored to mitigate these effects.^[22,23]^

### 2.8. Interaction with cancer stem cells

Emerging evidence suggests that neutrophils interact with cancer stem cells, a subpopulation of tumor cells with high plasticity and resistance to therapy. Neutrophils enhance CSC survival and self-renewal through the secretion of interleukins such as IL-6 and IL-8, thereby contributing to tumor recurrence and therapy resistance. Experimental studies have demonstrated that blocking neutrophil-derived IL-8 reduces CSC populations in TNBC models, providing a potential avenue for therapeutic intervention.^[24]^

### 2.9. Neutrophils immunotherapy in breast cancer

Immunotherapy has revolutionized cancer treatment by harnessing the body’s immune system to combat malignancies. Within this framework, the role of neutrophils, traditionally viewed as transient defenders against infections, has garnered increasing attention for their dual potential in breast cancer therapy. Their diverse and plastic nature within the tumor microenvironment (TME) presents both opportunities and challenges in immunotherapy, marking them as pivotal players in the ongoing evolution of cancer treatment strategies.

### 2.10. Reprogramming TANs

TANs, polarized by the TME, often adopt a pro-tumorigenic (N2) phenotype, promoting tumor progression, angiogenesis, and immune suppression. Reprogramming these TANs toward an anti-tumorigenic (N1) phenotype has emerged as a promising therapeutic approach. Preclinical studies have demonstrated that agents like TGF-β inhibitors can shift TANs to an N1 phenotype, enhancing their ability to recruit and activate cytotoxic T cells. For instance, in murine breast cancer models, blocking TGF-β signaling not only reprogrammed TANs but also amplified the efficacy of immune checkpoint inhibitors, such as anti-PD-1 and anti-CTLA-4 therapies. These findings suggest that combining TAN reprogramming with established immunotherapies could potentiate anti-tumor responses.^[25,26]^

### 2.11. Targeting neutrophil recruitment and trafficking

Neutrophils are recruited to the tumor site via chemokines, such as CXCL1, CXCL2, and IL-8, which interact with their corresponding receptors (e.g., CXCR2) on neutrophils. Therapeutic strategies aimed at disrupting this recruitment process have shown promise in limiting tumor growth and metastasis. CXCR2 antagonists, currently under clinical investigation, have demonstrated efficacy in preclinical breast cancer models by reducing neutrophil infiltration and restoring the activity of effector immune cells like T cells and NK cells. However, balancing this approach with the essential immune roles of neutrophils remains a critical challenge to prevent unintended immunosuppression.^[10,27]^

### 2.12. NETs as therapeutic targets

NETs, complex web-like structures released by activated neutrophils, play a significant role in metastasis by capturing circulating tumor cells and facilitating their colonization at distant sites. The therapeutic targeting of NETs is a burgeoning area of research. DNase enzymes, which degrade NETs, have shown potential in preclinical studies to reduce metastatic spread in breast cancer models. Additionally, NET inhibitors, such as PAD4 inhibitors, are being explored for their ability to prevent NETosis, thereby mitigating metastasis. Importantly, such approaches need to preserve the protective role of NETs in pathogen defense, requiring precise modulation of their activity.^[28,29]^

### 2.13. Combination therapies and synergy

Integrating neutrophil-targeted therapies with existing immunotherapies offers a synergistic strategy to enhance treatment outcomes in breast cancer. For example, reprogramming TANs to an N1 phenotype has been shown to enhance the efficacy of immune checkpoint inhibitors. Similarly, targeting NETs in combination with anti-angiogenic therapies could disrupt both metastatic pathways and the tumor’s blood supply, providing a dual-pronged attack on tumor progression. Personalized approaches, guided by biomarkers such as the NLR, are being explored to identify patients who are most likely to benefit from such combination therapies.^[30–32]^

### 2.14. Comparative overview of neutrophil activity across breast cancer subtypes

Breast cancer is a heterogeneous disease encompassing biologically distinct subtypes, including luminal A, luminal B, HER2-enriched, and TNBC. Each subtype displays unique molecular characteristics, clinical outcomes, and interactions with the immune microenvironment. While the role of neutrophils has been predominantly studied in TNBC due to its immunogenic nature and lack of targeted therapies, emerging evidence indicates that neutrophils also play subtype-specific roles in tumor progression and immune modulation.

#### 2.14.1. Luminal A and B subtypes (hormone receptor positive)

Luminal A and B tumors are characterized by the expression of estrogen receptor (ER) and/or progesterone receptor, with luminal B typically demonstrating higher proliferative indices and worse outcomes compared to luminal A. These subtypes generally exhibit a less inflamed tumor microenvironment, with lower levels of tumor-infiltrating lymphocytes and neutrophils. However, elevated peripheral NLR in luminal breast cancer patients has been associated with poorer survival outcomes, suggesting that systemic neutrophil activation may contribute to tumor progression even in these immunologically “cold” tumors. Moreover, neutrophil recruitment in luminal tumors may be facilitated by tumor-derived granulocyte-colony stimulating factor (G-CSF) and IL-8, promoting a shift toward the pro-tumorigenic N2 phenotype. The impact of neutrophils in this setting may involve endocrine resistance through oxidative stress and cytokine-mediated interference with hormone signaling.^[33]^

#### 2.14.2. HER2-enriched subtype

HER2-positive breast cancers overexpress the human epidermal growth factor receptor 2 and have benefitted from targeted therapies such as trastuzumab and pertuzumab. Interestingly, HER2 signaling may influence immune cell recruitment, including neutrophils. Preclinical studies suggest that HER2-overexpressing tumors secrete chemotactic factors like CXCL1 and CXCL8, which can attract neutrophils to the tumor site. In this context, neutrophils can exhibit dual roles. On one hand, ADCC triggered by anti-HER2 therapies may recruit and activate neutrophils as effector cells. On the other hand, a neutrophil-dominant microenvironment enriched with immunosuppressive factors such as arginase-1 and ROS may dampen the therapeutic efficacy of HER2-targeted immunotherapy.^[33]^

#### 2.14.3. Triple-negative breast cancer (TNBC)

TNBC lacks ER, progesterone receptor, and HER2 expression and is associated with high histologic grade, aggressive clinical behavior, and limited treatment options. It is the most immunogenic subtype and exhibits the highest levels of tumor-infiltrating lymphocytes, myeloid-derived suppressor cells (MDSCs), and TANs. Notably, TANs in TNBC are frequently polarized toward the N2 phenotype, promoting tumor growth, metastasis, and immune evasion. NET formation is particularly prominent in TNBC and has been implicated in pre-metastatic niche formation and resistance to ICB. Elevated NLR has also been strongly associated with poor response to ICB and chemotherapy in TNBC patients, further underscoring the immunosuppressive role of neutrophils in this setting.^[33]^

### 2.15. NLR: a composite marker of immune dysregulation

The NLR has emerged as a readily accessible and cost-effective prognostic marker in cancer, including breast cancer. However, its biological underpinnings extend beyond simple counts of circulating immune cells. A high NLR often reflects a shift in immune homeostasis, characterized by increased neutrophil-driven inflammation and concurrent lymphopenia, both of which contribute to impaired anti-tumor immunity.^[34]^ Neutrophils, particularly when activated by tumor-derived signals such as G-CSF, IL-6, and CXCL8, can accumulate systemically and within the tumor microenvironment. These cells can adopt an immunosuppressive phenotype (e.g., N2-like TANs or granulocytic MDSCs) that facilitates tumor progression through various mechanisms: suppression of cytotoxic T lymphocyte (CTL) activity via arginase-1 and ROS, promotion of angiogenesis, and support of metastatic dissemination. Thus, elevated neutrophil levels in peripheral blood may mirror the tumor’s ability to systemically reprogram the immune landscape in its favor.^[35]^

In contrast, lymphopenia, particularly a reduction in CD8⁺ T cells and other effector subsets, is associated with weakened immune surveillance and reduced responsiveness to immunotherapy. Chronic inflammation, tumor-derived factors, or chemotherapy-induced immunosuppression may contribute to decreased lymphocyte proliferation, apoptosis, or trafficking dysfunction. Therefore, a low lymphocyte count in the context of high neutrophil levels may indicate not just numerical imbalance, but functional immune collapse.^[34]^ Importantly, NLR should be viewed as more than a static metric; it represents a dynamic interplay between the innate and adaptive arms of the immune system. A rising NLR during treatment may signal immunotherapy resistance or systemic inflammation, whereas a decreasing NLR may reflect restoration of immune competency. Moreover, some studies suggest that integrating NLR with other immunologic or inflammatory biomarkers – such as platelet counts, C-reactive protein, or tumor-infiltrating lymphocyte profiles – could enhance its prognostic and predictive power.^[35]^

### 2.16. Conflicting evidence and nuances in neutrophil function and prognostic value

While a high NLR is frequently associated with poor prognosis across various cancers, including breast cancer, its interpretation is not universally straightforward. Several studies report that elevated NLR correlates with disease progression, reduced overall survival, and diminished response to therapies, particularly in TNBC. However, other investigations suggest that the prognostic value of NLR may vary with disease stage, treatment context, and immune milieu. For example, in early-stage tumors, neutrophils may exhibit anti-tumor properties by enhancing cytotoxic T cell responses, mediating ADCC, or directly attacking tumor cells via ROS and granule enzymes.^[33]^ This functional plasticity of neutrophils introduces complexity into therapeutic strategies. Some studies have documented beneficial roles of tumor-infiltrating neutrophils in early breast cancer subtypes, particularly when skewed toward an N1-like phenotype. These anti-tumor neutrophils may promote immune surveillance and contribute to effective tumor control. Conversely, in advanced or immunosuppressive tumor microenvironments, neutrophils often transition toward a pro-tumor (N2-like) phenotype, fostering angiogenesis, immune evasion, and metastasis. The timing and nature of neutrophil activity thus appear context-dependent, making them both allies and adversaries in cancer progression.^[34]^ Further complicating the narrative is the growing debate over the role of NETs in ICB efficacy. While some preclinical models suggest that NETs promote immunosuppression and impede T cell infiltration – making NET inhibition a promising strategy to enhance ICB response – other studies caution that disrupting NETs may impair antigen presentation or dampen innate immune signaling critical to immunotherapy success. This divergence points to a delicate immunological balance in which NETs may have both detrimental and potentially supportive roles, depending on tumor context, timing of therapy, and combination regimens.^[35]^

### 2.17. Challenges in neutrophils for breast cancer immunotherapy

Neutrophils have increasingly been recognized as pivotal players in cancer immunotherapy, especially in the context of breast cancer. While their potential to serve as therapeutic targets is promising, several challenges complicate their role in immunotherapy, making it difficult to harness their full potential for treatment. These challenges arise from the complexity of neutrophil behavior, their dual roles in cancer progression, and the dynamic tumor microenvironment (TME). Addressing these challenges is crucial for the successful development of neutrophil-based therapies.

#### 2.17.1. Dual functional roles of neutrophils

Neutrophils possess remarkable plasticity, enabling them to adopt either pro-tumorigenic or anti-tumorigenic roles depending on the signals present in the TME. Under normal circumstances, neutrophils function as part of the innate immune response, effectively fighting infections and promoting tissue repair. However, in the tumor microenvironment, they are often reprogrammed into TANs, which frequently adopt a pro-tumorigenic (N2) phenotype. In this state, neutrophils can promote tumor growth, metastasis, angiogenesis, and even immune suppression by secreting cytokines and chemokines that facilitate immune escape mechanisms. This dual nature of neutrophils presents a major challenge in designing therapies aimed at shifting neutrophils from their immunosuppressive role back to an anti-tumor phenotype. Reprogramming TANs to an anti-tumorigenic (N1) state has shown promise in preclinical studies, but the complexity of regulating this shift in a tumor-specific manner remains a major hurdle. The tumor’s ability to continuously alter the immune environment means that neutrophils could be manipulated back into a pro-tumor state despite therapeutic interventions. Furthermore, the fact that neutrophils contribute to both tumor progression and defense against pathogens complicates the process of selectively targeting their cancer-related functions without interfering with their essential immune surveillance activities.^[36]^

#### 2.17.2. Heterogeneity of breast cancer subtypes

Breast cancer is a highly heterogeneous disease, with different subtypes such as hormone receptor positive, HER2-positive, and TNBC presenting distinct biological behaviors, responses to treatment, and immunological landscapes. This diversity is reflected in the varying roles neutrophils play across different breast cancer subtypes. In some cases, neutrophils may promote tumor aggression and resistance to therapies, particularly in TNBC, which is often more aggressive and less responsive to conventional treatments like hormonal therapy or HER2-targeted therapy. Conversely, in other subtypes, neutrophils may play a more benign role or even contribute to anti-tumor immunity. The challenge, therefore, is to develop neutrophil-targeted therapies that are both subtype-specific and adaptable to the heterogeneity of the disease. It is crucial to understand how neutrophils interact with other immune cells and tumor components in each specific breast cancer subtype, as this could influence the success of any immunotherapeutic approach. A one-size-fits-all approach is unlikely to be effective, and personalized strategies based on the unique immune profile of each breast cancer patient are needed.^[37]^

#### 2.17.3. Tumor microenvironment and immunosuppressive signals

The TME is rich in immunosuppressive signals, including cytokines like TGF-β and IL-10, which can drive neutrophils into a pro-tumorigenic phenotype, exacerbating tumor progression. The TME also includes other immune-suppressive cells, such as regulatory T cells (Tregs) and MDSCs, which can further modulate neutrophil behavior. The complex interplay between neutrophils and these suppressive factors presents a significant challenge to neutrophil-targeted immunotherapy. For instance, while certain therapies aim to reprogram TANs to enhance their anti-tumor functions, the surrounding immunosuppressive signals can neutralize these efforts, leading to minimal therapeutic benefit. Additionally, the tumor’s ability to adapt and produce immunosuppressive molecules means that the reprogramming of neutrophils alone may not be sufficient to trigger a sustained anti-tumor response. Overcoming these immunosuppressive signals requires a multifaceted approach that targets both neutrophils and the broader TME.^[38]^

#### 2.17.4. Neutrophil recruitment and selective targeting

Neutrophils are recruited to tumors through a series of signaling molecules, including chemokines like CXCL1, CXCL2, and IL-8. Targeting these pathways to reduce neutrophil infiltration may hold promise in preventing tumor progression and metastasis. However, neutrophils also play an essential role in host defense, particularly in fighting infections. As such, interfering with their recruitment to the tumor could have unintended consequences, such as increasing the risk of infections or impairing the body’s ability to fight off pathogens. Furthermore, the selectivity of neutrophil-targeted therapies is a key challenge. While reducing neutrophil infiltration into tumors may slow cancer progression, it is critical that therapies are carefully designed to avoid detrimental effects on systemic immunity. Balancing the inhibition of neutrophil recruitment to tumors while preserving their role in immune surveillance remains a difficult task.^[39]^

#### 2.17.5. NETs and tumor metastasis

NETs, which are released by activated neutrophils, have been shown to promote tumor metastasis by capturing and facilitating the extravasation of circulating tumor cells. While targeting NETs has shown potential in preclinical models, particularly in reducing metastatic spread, this strategy presents its own set of challenges. NETs also serve an essential immune function by trapping pathogens, and their inhibition could impair the body’s ability to defend against infections.^[40]^

Despite the promise of neutrophil-targeted strategies in augmenting cancer immunotherapy, several critical challenges must be addressed before clinical translation can be realized. One of the foremost concerns is the potential for systemic immunosuppression, as neutrophils play an essential role in host defense against bacterial and fungal pathogens. Targeting neutrophil recruitment, activation, or survival – especially through agents like CXCR2 antagonists or neutrophil elastase inhibitors – may inadvertently increase infection susceptibility, particularly in immunocompromised cancer patients. Additionally, neutrophil-modulating agents may lead to off-target effects and systemic toxicity, including hematologic imbalances and impaired wound healing. Therefore, patient stratification based on tumor neutrophil profiles, peripheral NLRs, or specific immune gene signatures will be essential to identify individuals most likely to benefit from such interventions. Furthermore, the development of robust and predictive biomarkers is urgently needed to monitor therapeutic responses and guide treatment decisions. Long-term safety data from prospective clinical trials must also be established to ensure that modulation of neutrophil function does not compromise overall immune competence or induce unintended pro-tumor effects. Addressing these risks through thoughtful clinical design and mechanistic studies will be key to safely harnessing neutrophils in the immunotherapy landscape of breast cancer.^[33–35]^

### 2.18. Recommendations for neutrophil-based immunotherapy in breast cancer

Given the complexities surrounding the role of neutrophils in breast cancer, it is crucial to develop strategies that not only harness their potential but also address the challenges associated with their manipulation. Below are key recommendations to guide future research and clinical efforts in neutrophil-based immunotherapy for breast cancer.

#### 2.18.1. Personalized approaches based on tumor subtype

Considering the heterogeneity of breast cancer and the varying roles neutrophils play in different subtypes, a personalized approach to neutrophil-targeted immunotherapy is essential. Research should focus on developing subtype-specific strategies that consider the distinct immune landscapes of hormone receptor positive, HER2-positive, and TNBC. Biomarker-driven therapies that profile neutrophil activity in individual patients could enable the development of more tailored treatments. Personalized medicine, which includes analyzing tumor microenvironmental factors such as neutrophil infiltration, cytokine profiles, and immune cell interactions, could allow for more effective neutrophil reprogramming and immunotherapy outcomes.

#### 2.18.2. Targeting neutrophil reprogramming and plasticity

The plasticity of neutrophils – allowing them to switch between pro-tumorigenic and anti-tumorigenic phenotypes – presents a critical challenge in immunotherapy. Therefore, future therapeutic strategies should focus on selectively reprogramming neutrophils toward an anti-tumorigenic (N1) phenotype in the context of breast cancer. Research should explore innovative approaches, such as small molecule inhibitors, monoclonal antibodies, or gene therapies, to modulate neutrophil polarization. Understanding the signaling pathways that drive neutrophil polarization, such as the CXCR2 to CXCL8 axis, could help design therapies that selectively guide neutrophils to adopt a tumor-suppressive role without impairing their overall immune function.

#### 2.18.3. Combination therapies with other immunomodulatory agents

Neutrophil-targeted therapies are likely to be more effective when combined with other immunotherapeutic strategies. For example, combining neutrophil modulation with immune checkpoint inhibitors (such as PD-1/PD-L1 blockers) could enhance the anti-tumor immune response. Neutrophils, through their interactions with T cells, could help overcome the immunosuppressive effects of tumor-associated macrophages and regulatory T cells (Tregs), improving the efficacy of checkpoint blockade therapies. Additionally, combining neutrophil-targeted therapies with anti-angiogenic agents or cytotoxic drugs could help reduce tumor progression while reprogramming the TME in favor of anti-tumor immunity.

#### 2.18.4. Developing safe strategies to target NETs

The role of NETs in promoting metastasis suggests that targeting NET formation could be a promising therapeutic approach. However, as NETs play a vital role in immune defense, it is essential that therapies targeting NETs be designed carefully to avoid impairing the body’s ability to fight infections. Future research should focus on developing specific inhibitors of NET formation that do not compromise neutrophil functionality in pathogen defense. Additionally, studies should assess the systemic effects of NET inhibition, ensuring that such therapies do not result in a heightened risk of infections or other immune-related side effects.

#### 2.18.5. Enhancing understanding of neutrophil-tumor interactions

More research is needed to better understand how neutrophils interact with other immune cells and components of the TME, including tumor cells, stromal cells, and endothelial cells. Understanding the complex crosstalk between neutrophils and other immune cells such as macrophages, T cells, and dendritic cells will allow for the development of more effective immunotherapeutic strategies. Detailed studies on the role of neutrophils in promoting tumor-associated inflammation, angiogenesis, and immune evasion will inform the development of therapies that can disrupt these processes. Furthermore, tracking the dynamics of neutrophil behavior over time in response to various treatments could help refine therapeutic approaches.

#### 2.18.6. Addressing neutrophil heterogeneity in tumor models

The diversity of neutrophil phenotypes within the tumor microenvironment poses a challenge in understanding their exact role in tumor progression. Research should focus on developing more accurate preclinical models, such as humanized mouse models or organoids, to study neutrophil heterogeneity in breast cancer. These models could facilitate a deeper understanding of how different neutrophil subsets contribute to tumor growth, immune evasion, and metastasis. Additionally, advanced imaging techniques to monitor neutrophil behavior in real-time within tumors could provide valuable insights into their dynamic functions and guide therapeutic interventions.

#### 2.18.7. Monitoring neutrophil infiltration and function in clinical trials

To effectively translate neutrophil-based immunotherapy into clinical practice, it is essential to incorporate robust monitoring of neutrophil infiltration and function in clinical trials. Biomarkers for neutrophil activation, polarization, and NET formation should be included in clinical trial protocols to assess the efficacy of neutrophil-targeted therapies. Imaging techniques such as flow cytometry and immunohistochemistry can be used to track neutrophil distribution within tumors and correlate this data with clinical outcomes. Such monitoring will help determine the optimal timing, dosing, and combinations of neutrophil-targeted therapies, improving patient-specific treatment regimens.

#### 2.18.8. Managing potential side effects and toxicity

Given the essential role of neutrophils in immune defense, targeting these cells for cancer immunotherapy must be approached with caution to minimize potential side effects, including increased susceptibility to infections and systemic inflammation. Research should focus on developing therapies that selectively modulate neutrophil functions in the tumor microenvironment without compromising their innate immune roles. Moreover, clinical trials must evaluate the safety profile of neutrophil-targeted treatments, particularly in terms of infection risk, systemic inflammation, and other immune-related adverse events.

## 3. Conclusion

Neutrophils, once considered mere foot soldiers of the innate immune system, have emerged as critical players in breast cancer progression and potential targets for immunotherapy. Their dualistic nature – capable of both promoting and suppressing tumor growth – highlights their complexity and the need for a nuanced approach to therapeutic interventions. TANs and NETs have been identified as key mediators of tumor progression, immune suppression, metastasis, and therapy resistance. These findings underscore the potential of modulating neutrophil functions to enhance anti-tumor immunity while minimizing pro-tumor activities.

However, significant challenges remain in translating these insights into clinical practice. Neutrophil heterogeneity, phenotypic plasticity, and the risk of disrupting their critical roles in immune defense present barriers that must be carefully navigated. Furthermore, differences in neutrophil biology between humans and preclinical models complicate the design and success of clinical trials. Addressing these challenges will require precision medicine approaches that tailor therapies to individual patients, integrate neutrophil modulation with existing immunotherapies, and consider the unique biology of breast cancer subtypes.

## Author contributions

**Conceptualization:** Emmanuel Ifeanyi Obeagu.

**Methodology:** Emmanuel Ifeanyi Obeagu.

**Resources:** Emmanuel Ifeanyi Obeagu.

**Supervision:** Emmanuel Ifeanyi Obeagu.

**Validation:** Emmanuel Ifeanyi Obeagu.

**Visualization:** Emmanuel Ifeanyi Obeagu.

**Writing – original draft:** Emmanuel Ifeanyi Obeagu.

**Writing – review & editing:** Emmanuel Ifeanyi Obeagu.
